# Characterization of *ex vivo* expanded natural killer cells for cancer immunotherapy

**DOI:** 10.1111/imcb.70038

**Published:** 2025-06-18

**Authors:** Jin Young Min, Tae Kyung Ko, Hye Min Kim, Hae Won Jung, Cha Ok Yim, Eun Hee Han

**Affiliations:** ^1^ Biopharmaceutical Research Center, Ochang Institute of Biological and Environmental Science Korea Basic Science Institute (KBSI) Cheongju South Korea; ^2^ TSBIO ALL Co., Ltd Seoul South Korea; ^3^ Korea University of Science and Technology (UST) Daejeon South Korea; ^4^ College of Medicine Inha University Incheon Republic of Korea

**Keywords:** antibody‐dependent cellular cytotoxicity, donor, HER2, natural killer cells, peripheral blood mononuclear cells, solid cancer

## Abstract

In this study, we employed a coculture system to expand natural killer (NK) cells *ex vivo* from healthy donors and patients with breast cancer and investigated their surface marker expression. We further analyzed the activation markers of primary expanded NK cells on Day 13 using cytokine arrays and dimensionality reduction techniques. Cytokine profiles were observed on Days 0, 6 and 13 (TS*‐*NK). To validate the anticancer activity of the expanded NK cells, we conducted lactate dehydrogenase assays against the hematologic cancer cell line K562 using cells from 10 donors (five patients with cancer and five healthy individuals). Additionally, we examined the antibody‐dependent cellular cytotoxicity (ADCC) of differentiated NK cells cocultured with SK‐BR‐3 cells in the presence of the HER2‐targeting monoclonal antibodies, trastuzumab and pertuzumab. Our findings demonstrate the stable expansion of NK cells from donor peripheral blood mononuclear cells and their potent anticancer effects and ADCC against both hematologic and solid tumors, highlighting their potential as a versatile therapeutic approach in oncology.

## Graphical abstract

In this study, we characterized the phenotype and function of *ex vivo* expanded TS‐natural killer (NK) cells over a 13‐day culture period. We show dynamic cytokine secretion profiles and enhanced cytotoxicity upon coculture with tumor targets, supporting their utility in cancer immunotherapy.

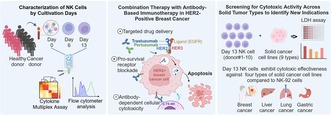

## INTRODUCTION

Natural killer (NK) cells are critical components of the body's initial immune defense and play a pivotal role in thwarting tumor development and metastasis.^1^, ^2^ These cells execute tumor‐eliminating functions through the release of cytokines and lytic granules, leveraging a unique advantage in cancer immunotherapy due to their ability to act independently of the major histocompatibility complex (MHC).^3^ This characteristic makes NK cells potent antitumor effectors, second only to T cells for promising oncological applications.^4^

To enhance their therapeutic potential, *in vitro* expansion of both autologous and allogeneic NK cells has emerged as a key strategy for treating various cancer types. Among these approaches, recent advancements in NK cell‐based therapies have explored multiple sources for NK cell generation, including cord blood, peripheral blood and induced pluripotent stem cell (iPSC)‐derived NK cells, each with distinct advantages and limitations.

Cord blood NK cells provide a valuable source of NK progenitors with reduced immunogenicity, making them a suitable candidate for allogeneic applications. However, their low yield and limited donor availability present challenges for clinical scalability. Peripheral blood NK cells are more readily accessible and commonly used in clinical applications, yet their large‐scale expansion remains a significant hurdle. Meanwhile, iPSC‐derived NK cells offer an unlimited source, allowing for the generation of standardized, off‐the‐shelf products. However, their high production costs and stringent regulatory requirements may limit widespread clinical adoption.^5^, ^6^

Given these considerations, our study focuses on optimizing the expansion and functional characterization of peripheral blood‐derived NK cells, which provide a practical balance between accessibility and clinical feasibility for immunotherapeutic applications. To successfully translate NK cell therapy into clinical practice, however, it remains crucial to secure a sufficient quantity of highly purified NK cells to achieve optimal therapeutic outcomes. This is particularly true for both autologous and allogeneic NK cell therapies, where the large‐scale expansion of donor NK cells is imperative to secure a sufficient number of high‐purity activated NK cells. Various strategies for the *in vitro* expansion of clinical‐grade NK cells have been explored, including the differentiation of NK cells from sources such as umbilical cord blood and PBMCs derived from either whole blood or leukapheresis.^7^, ^8^, ^9^ Among these, leukapheresis is frequently preferred for PBMC collection because of its sterile, closed system, making it ideal for Good Manufacturing Practice (GMP)‐compliant cultivation of NK cells.^7^, ^10^, ^11^

NK cells undergo differentiation and maturation to form distinct subpopulations: CD56^bright^CD16^dim^ and CD56^dim^CD16^bright^.^12^, ^13^ The former predominantly secretes chemokines and cytokines, which are vital for orchestrating the immune response, whereas the latter is more directly involved in cytotoxic activities through the release of perforin and granzyme A/B.^14^ Importantly, NK cell antibody‐dependent cellular cytotoxicity (ADCC), a key tumor‐killing mechanism, is mediated by CD16, highlighting its broad antitumor activities, including interactions between the Fas/Fas ligand and cytokine‐mediated effects.^15^ ADCC is a critical immune response mechanism in which antibodies, by binding to their target antigens on tumor cells, flag these cells for destruction by immune effector cells, such as NK cells, through the engagement of the CD16 receptor. This process is fundamental to the efficacy of monoclonal antibody therapies such as trastuzumab and pertuzumab, which target cancer cells for destruction by the immune system.

In the context of breast cancer and other human epidermal growth factor receptor 2 (HER2)‐positive tumors, enhancing NK cell efficacy and ADCC represent critical methods for improving the outcomes of targeted therapies. Trastuzumab and pertuzumab, both humanized monoclonal antibodies targeting HER2, underscore the indispensable role of the immune system in mediating antitumor effects.^16^ These therapies involve NK cells via CD16, which facilitate a powerful ADCC response.^17^ However, the efficacy of these treatments is hampered in patients with breast cancer because of the altered expression of activating receptors on NK cells, which substantially reduces their cytotoxic potential.^16^, ^18^

Despite significant advancements in NK cell therapies, challenges such as limited NK cell expansion and inconsistent anticancer efficacy remain. This study aimed to address these by providing a detailed analysis of the ADCC and anticancer effects of donor‐derived NK cells, which are crucial for improving clinical outcomes in cancer therapy. Given this challenge, our study aimed to rigorously assess the ADCC and anticancer effects of donor‐derived NK cells, using a coculture system of HER2‐positive breast cancer cells and an array of other solid tumor cells. By analyzing the cytotoxicity of NK cells against a diverse set of cancer types, we aimed to validate their utility and enhance our understanding of NK cell‐based therapies, reinforcing their potential to revolutionize cancer treatment. Our findings contribute to the growing body of evidence supporting the enhancement of NK cell functionality as a strategic approach for augmenting the efficacy of HER2‐targeted therapies.

Through this study, we reaffirmed the critical function of NK cells in oncology, particularly in harnessing their innate ability to combat solid tumors through ADCC and other mechanisms. Our findings contribute to a growing body of evidence supporting the enhancement of NK cell functionality as a strategic approach for augmenting the efficacy and sensitivity of current and future HER2‐targeted therapies.

## RESULTS

### Enhanced purity and expansion of NK cells for cancer therapy: a good manufacturing practice (GMP)‐compliant approach

**Figure 1:**
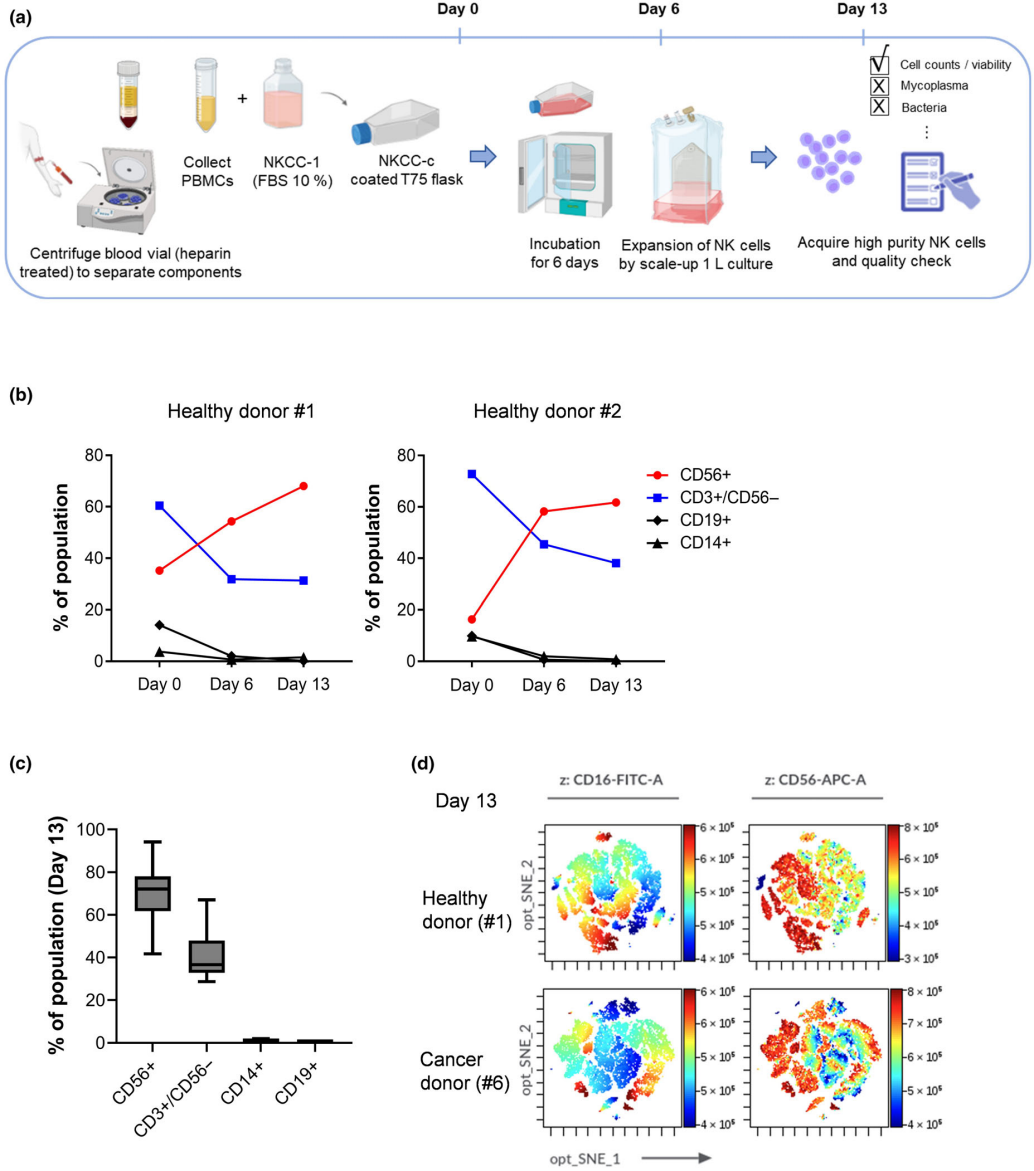
Analysis of the phenotype and purity of NK cells expanded during a 13‐day culture period under good manufacturing practice (GMP) conditions. **(a)** Schematic representation of the NK cell expansion process used in this study. NK cells were cultured for 13 days in a CO_2_ incubator using an NK expansion kit supplemented with various growth factors and cytokines. The process was divided into three key stages: PBMC isolation (Day 0), activation phase (Day 6) and expansion phase (Day 13). **(b)** Flow cytometry analysis of peripheral blood mononuclear cells (PBMCs) from two donors, illustrating the percentage of cells expressing CD56^+^ (NK cells), CD3^+^ (T cells), CD14^+^ (monocytes) and CD19^+^ (B cells) markers at baseline (Day 0), mid‐expansion (Day 6) and at the end of the culture period (Day 13). **(c)** Consolidated plot representing the proportions of the same cell markers in the expanded NK cell populations across all 10 donors by Day 13, providing insight into the consistency of the expansion process and the relative enrichment of NK cells. **(d)** Dimensionality reduction plot (opt‐SNE) generated using CytoBank software, visualizing the phenotypic landscape of the CD16^+^ and CD56^+^ populations within the Day 13 expanded NK cells from one cancer patient donor and one healthy donor, allowing for the comparison of NK cell phenotypes between different sources. This figure underscores the effectiveness of the GMP‐compliant expansion protocol in selectively proliferating NK cells while maintaining their key functional markers.

A GMP‐compliant NK cell culture system was established to enable the large‐scale expansion and purification of NK cells for therapeutic applications. As illustrated in Figure 1a, the process consists of three key phases: isolation of peripheral blood mononuclear cells (PBMCs) from donor blood (Day 0), an initial activation and culture phase using NKCC‐c coated flasks (Days 0–6), and a large‐scale expansion phase (Days 6–13). On Day 13, the expanded NK cell population underwent a series of quality assessments to ensure high purity, viability, and the absence of contaminants such as mycoplasma and bacteria.

To assess the yield and proliferation efficiency of the NK cell culture system, total cell counts were measured at different time points (Days 0, 6 and 13) across multiple donor samples (Supplementary figure 1a). By Day 6, the total cell count increased approximately 6.3‐fold, and by Day 13, the NK cell population expanded by an average of 32.6‐fold, demonstrating the effectiveness of the culture protocol (Supplementary figure 1b). While this expansion is sufficient for research‐scale applications, it is notably lower than the 1000‐fold expansion typically observed in large‐scale GMP bioreactors (50 L systems). This difference highlights key bioprocess factors affecting NK cell proliferation efficiency, including oxygen/nutrient availability, density‐dependent growth limitations and metabolite accumulation. These aspects are further examined in the Discussion section, underscoring the importance of bioprocess optimization beyond simple volumetric scaling.

Flow cytometric analysis conducted on the immune cell phenotypes of PBMCs (Day 0) and NK cells (Day 6 or 13) from two donors revealed that NK cells differentiated by TSBio on Day 13 exhibited a significant reduction in the population of CD14^+^ monocytes, CD19^+^ B cells and CD3^+^/CD56^−^ cells compared to that at Day 0 PBMC composition (Figure 1b). Conversely, there was a notable increase in the population of CD56^+^ cells, indicating the successful enrichment and purity enhancement of the NK cell product (Figure 1b). This outcome underscores the effectiveness of TSBio's GMP‐compliant processes in achieving high‐purity expanded NK cells, thus setting a benchmark for the application of NK cell therapies in cancer treatment.

During flow cytometry analysis, isotype controls were included to establish proper gating thresholds for immune cell populations (Supplementary figure 1c). This gating strategy ensured precise identification of CD16^+^, CD56^+^, CD3^+^, CD14^+^ and CD19^+^ subsets in the expanded NK cell population.

Upon analyzing NK cells from all 10 donors on Day 13, the population of CD56^+^ cells was approximately 75%, demonstrating significant enrichment of NK cells. The percentage of cells that did not express CD56 but expressed CD3 was approximately 35%. Moreover, the populations of CD14^+^ monocytes and CD19^+^ B cells were significantly reduced, each constituting < 1% of the cell population, highlighting the efficiency of the NK cell expansion process in achieving high‐purity NK cells, as illustrated in Figure 1c. The decision to not remove CD3^+^ T cells during the NK cell expansion process was based on the following considerations. Firstly, the presence of CD3^+^ T cells can potentially provide supportive signals and cytokines that may enhance NK cell proliferation and activation. Additionally, the interactions between NK cells and CD3^+^ T cells in the coculture system can more accurately mimic the physiological environment where various immune cells interact and cooperate.^19^, ^20^

Subsequently, the composition of the CD16^+^ cell population within the CD56^+^ cell subset of expanded Day 13 NK cells, produced by TSBio, was explored using advanced dimensionality reduction. An opt‐SNE plot, generated using CytoBank software, facilitated the visualization of this specific cell subset. The analysis included samples from both a healthy donor and a breast cancer patient, allowing for a direct comparison of CD16^+^ cells within the broader CD56^+^ NK cell population. The representative opt‐SNE plots from one healthy donor and one cancer patient in Figure 1d highlight distinct yet overlapping NK cell phenotypic landscapes. While Figure 1d provides an illustrative example of individual donor variability, Supplementary figure 1d expands upon this by presenting data from multiple donors, reinforcing the robustness and generalizability of our observations.

To assess potential differences in NK cell phenotype between healthy and cancer donors after expansion, we analyzed the ratio of CD56^+^/CD16^+^ NK cells using flow cytometry (Supplementary figure  1e). NK cells from healthy donors exhibited a trend toward higher CD16 expression, though this difference was not statistically significant. Overall, these findings indicate that NK cell expansion did not result in significant phenotypic or functional differences between cells derived from healthy and cancer donors.

### Dynamic cytokine expression profiles in NK cell cultivation: Enhancing therapeutic potential through optimized culture conditions

**Figure 2:**
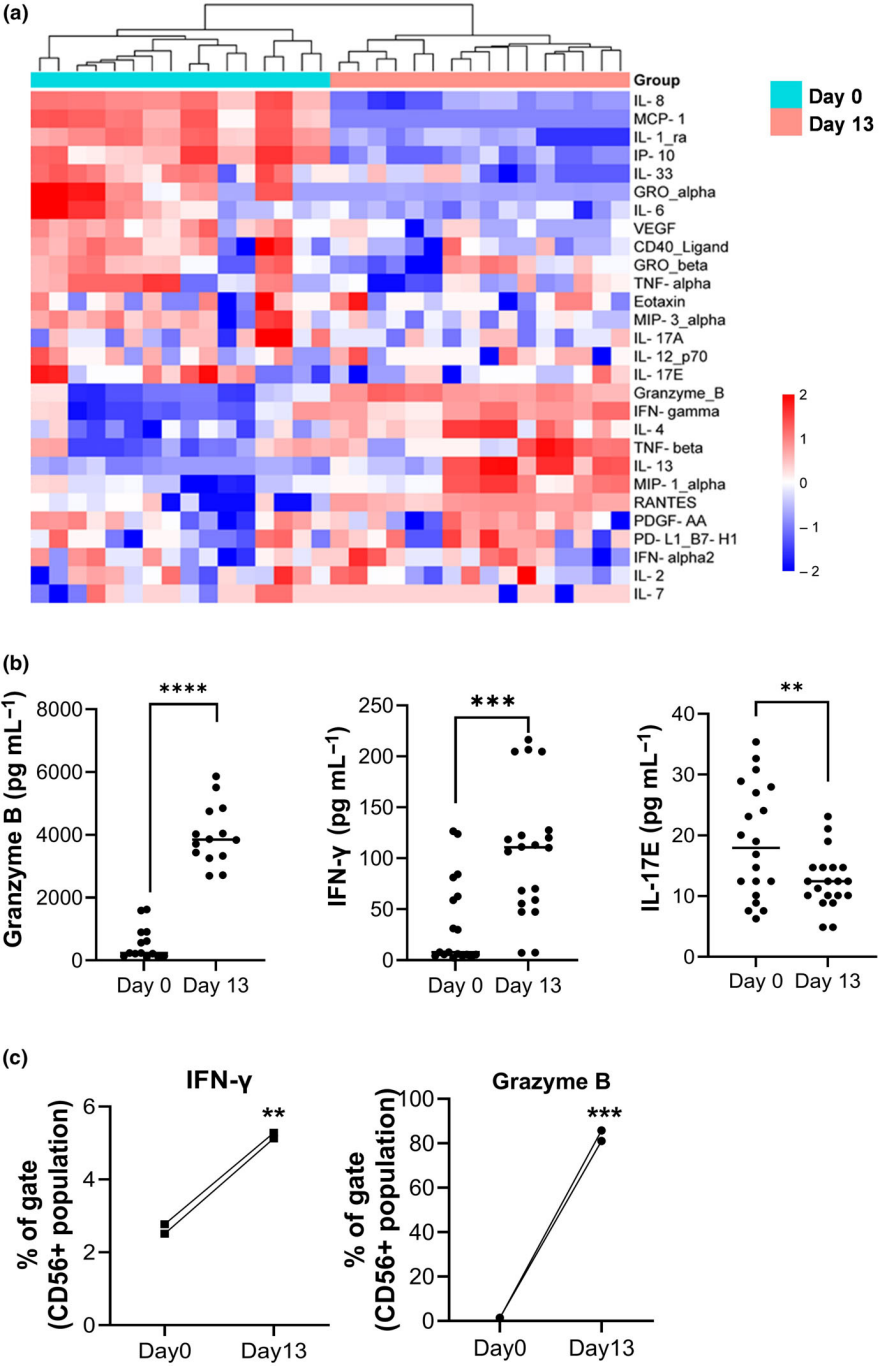
Cytokine dynamics in the culture medium of NK cells expanded from 10 donors over a 13‐day period. **(a)** Heatmap analysis of cytokine profiles at Days 0 and 13, excluding samples with undetectable values. Data were analyzed in duplicate and subjected to hierarchical clustering based on the concentration levels of 28 distinct cytokines. The clustering pattern demonstrates the segregation of samples into distinct groups, reflecting shifts in cytokine secretion profiles as NK cells progress from an early culture state to an expanded, functionally enhanced state at Day 13. Corresponding *P*‐values are provided in Table 1. **(b)** Graph highlighting the cytokines with the most significant changes on Day 13 relative to Day 0, specifically focusing on granzyme B, IFN‐γ, and IL‐17E, indicating a marked enhancement in the cytotoxic and inflammatory potential of the cultured NK cells at the conclusion of the expansion process. **(c)** Comparison of cytokine secretion from NK cells (CD56+ population) at Day 0 and Day 13 from two representative healthy donors (#1, #2). Flow cytometry analysis confirmed that IFN‐γ and granzyme B secretion significantly increased by Day 13, demonstrating that expanded NK cells are the primary source of these cytokines. These results support the functional enhancement of NK cells following expansion, reinforcing their therapeutic potential. Statistical significance is indicated as follows: **P < 0.01, ***P < 0.001, ****P < 0.0001.

Our objective was to ascertain whether optimally cultivated NK cells exhibiting superior therapeutic attributes for cancer treatment could be achieved by mapping cytokine expression trends against the stages of NK cell maturation. By analyzing the cytokine expression patterns across 10 donors, we identified distinct expression profiles for 28 cytokines, showing significant variations across the culture periods of NK cells (Days 0 and 13) irrespective of the donor's health status. These patterns were methodically illustrated using hierarchical cluster analysis (Figure 2a, Table 1), where the intensity of cytokine expression was represented by a color gradient.

**Table 1:** Average concentrations (pg mL^−1^) of 28 cytokines in culture supernatants across different timepoints and co‐culture conditions.

#	Analyte	Mean ± SD			
		Day 0	Day 6	Day 13	Day 13 + SK‐BR‐3
1	CCL2/JE/MCP‐1	510.2 ± 334.9	17.8 ± 8.4	7 ± 0.1****	57.6 ± 56***
2	CCL3/MIP‐1 alpha	167.7 ± 82.1	1687 ± 1327.7	425.9 ± 203.6	778.4 ± 218.3***
4	CCL5/RANTES	210.5 ± 251.9	3059.6 ± 1652.4	1824.2 ± 887.3****	2300.3 ± 925.2
5	CCL11/Eotaxin	14.6 ± 1.2	16.2 ± 2.3	14.7 ± 1.1	15.1 ± 0.9
7	CCL20/MIP‐3 alpha	7.4 ± 3.1	12.9 ± 5.5	5.1 ± 1.3***	8 ± 1.6****
8	CD40 Ligand/TNFSF5	738.1 ± 194.8	1240.2 ± 656.1	600.8 ± 134.7**	694.2 ± 149.6
9	CXCL1/GRO alpha/ KC/CINC‐1	141.8 ± 64.3	68.3 ± 1.6	66.7 ± 0.6***	67.9 ± 0.5***
10	CXCL2/GRO beta/ MIP‐2/CINC‐3	55.6 ± 23.7	73.6 ± 59.4	38.3 ± 26.4	76.3 ± 31**
11	CXCL10/IP‐10/CRG‐2	27.6 ± 22.9	25.7 ± 31.2	1 ± 0.7****	11.6 ± 13.2***
17	Granzyme B	485.3 ± 516.9	251335.1 ± 258455.8	5274.4 ± 2758****	9265.3 ± 5171.2**
18	IFN‐alpha 2/IFNA2	2.4 ± 0.6	4.6 ± 2.3	2.6 ± 0.7	2.7 ± 0.7
20	IFN‐gamma	30.3 ± 41.8	3158.2 ± 3483.1	117.5 ± 58.6***	624.2 ± 260.3****
23	IL‐1ra/IL‐1F3	103.7 ± 55.2	57.6 ± 21.7	4.7 ± 3.2	8.6 ± 4.5*
24	IL‐2	21867.6 ± 1560.5	21744.5 ± 1237.6	22014.7 ± 1350.3	20489.9 ± 4843
26	IL‐4	0.5 ± 0.2	3.2 ± 3.4	1.1 ± 0.4**	1.4 ± 0.4*
27	IL‐5	5.8 ± 0.4	770.6 ± 657.7	21.6 ± 14.5	22.7 ± 16.3
28	IL‐6	10.2 ± 8.7	25.2 ± 24.9	3.9 ± 1**	6.8 ± 1.4****
30	IL‐8/CXCL8	1781.2 ± 1265.6	541.5 ± 595	15.2 ± 12****	27.1 ± 13.5*
33	IL‐12 p70	6.3 ± 2.9	18.2 ± 6.4	6.4 ± 3.1	8.8 ± 3.1
34	IL‐13	46.5 ± 4	548.9 ± 630.8	97 ± 44.4	144.5 ± 74.8*
36	IL‐17/IL‐17A	2.4 ± 1.2	2.7 ± 0.9	1.9 ± 0.4*	1.9 ± 0.5
37	IL‐17E/IL‐25	16.2 ± 8	19.5 ± 9.9	12.5 ± 4.8**	10.9 ± 5
38	IL‐33	7.1 ± 3.1	11.3 ± 5.5	3.2 ± 2.3****	4.2 ± 2.7
39	Lymphotoxin‐alpha/TNF‐beta	4.3 ± 2.6	134.9 ± 120.2	7.7 ± 4.2	11.4 ± 5.3*
40	PD‐L1/B7‐H1	2.2 ± 1.4	7.3 ± 5	2.9 ± 1.8	4.4 ± 2.2
41	PDGF‐AA	7.5 ± 4	12.5 ± 7.7	7.3 ± 4.5	13.8 ± 4.4***
44	TNF‐alpha	73.6 ± 45.4	121.5 ± 160	30.3 ± 21.3***	286.1 ± 217.2****
46	VEGF	3.8 ± 1.7	12.1 ± 6	1.7 ± 0.9**	13.2 ± 6.3****

*P*‐values indicate statistical comparisons between Day 13 *versus* Day 0 and Day 13 *versus* Day 13 + SK‐BR‐3.

* *P* < 0.05.

** *P* < 0.05.

*** *P* < 0.01.

**** *P* < 0.001.

Following a comprehensive analysis of cytokine expression trends, 28 cytokines exhibited significant variations across the three key stages of NK cell culture (Days 0, 6 and 13) (Supplementary figure 2a).

To further confirm these changes, Principal Component Analysis (PCA) was conducted to evaluate overall shifts in cytokine expression patterns (Supplementary figure 2b). The PCA results revealed distinct clustering of samples based on culture days, indicating a progressive shift in cytokine dynamics during NK cell expansion. Statistical analysis confirmed significant differences in cytokine profiles between Day 0 and Day 13 (ANOVA, *P* < 0.0001), reinforcing substantial alterations in cytokine secretion throughout the culture period (Figure 2a).

Notably, among these cytokines, IL‐2, a crucial component added to the culture medium to sustain NK cell viability, was prominently expressed on Days 0 and 13. Moreover, the levels of granzyme B and IFNγ, which are hallmark indicators of NK cell activity, alongside cytokines such as IL‐17E, known to promote expression, were markedly elevated on Day 13 compared to those on Day 0 (Figure 2b). This cytokine profile suggests that NK cells expanded from Day 0 (PBMCs) to Day 13 through TSBio's manufacturing process and exhibited heightened activity characteristics, indicating their enhanced potential for cancer therapy. However, while the increase in cytokine secretion was evident, it was necessary to confirm that these cytokines were primarily derived from NK cells rather than other immune cell populations present in the culture. To address this, we performed flow cytometry analysis of cytokine production specifically within the CD56^+^ NK cell population at different time points. The results confirmed a significant increase in NK‐derived cytokine production by Day 13, with IFN‐γ levels approximately doubling and Granzyme B levels increasing 62‐fold relative to Day 0. Notably, while analyzing the cytokine profiles, we observed an apparent increase in IL‐33 levels in Day 13 samples on the heatmap (Figure 2a). However, this is a visualization artifact due to z‐score normalization across donors. The raw cytokine values (Supplementary figure 2a, Table 1) confirm that IL‐33 levels actually decreased significantly from Day 0 to Day 13 across all donors. As IL‐33 is a stromal‐derived cytokine and not typically secreted by NK cells, its presence at early time points likely reflects residual non‐NK cells in PBMCs. This signal diminishes as NK cells become enriched. These findings provide direct evidence that expanded NK cells are the primary source of these cytokines, reinforcing their functional potential for therapeutic applications (Figure 2c).

### Multifaceted analysis of NK cell activation and tumor invasion potential

To elucidate the activation status of NK cells, which are pivotal for efficacious cancer immunotherapy and their capability to navigate and penetrate the tumor milieu, we utilized flow cytometry with two distinct panel types. Detailed characterization of surface markers and cytokine profiles provides insights into the mechanisms enhancing NK cell‐mediated cytotoxicity, potentially translating to more effective cancer therapies. For example, the expression of CD69 and CD62L indicates NK cells' readiness for activation and tumor infiltration, which are critical for their anticancer efficacy.

*NK* activation/chemokine receptor panel flow cytometry analysis of Day 13 NK cells, visualized through a dimension‐reduced opt‐SNE plot, revealed that CD62L and CD69 markers were coexpressed within the CD56^+^ cell population (Supplementary figure 3a). Given the established functions of CD69 and CD62L, it was inferred that the Day 13 NK cells exhibit a phenotype conducive to immune activation and directed migration toward lymphoid structures within the CD56^+^ NK cell subset.

Furthermore, analysis of the NK activation receptor panel revealed expression of CD94, CD160, CD244, CD226 (DNAM‐1), CD314 (NKG2D) and CD336 (NKp44) within the CD56^+^ population (Supplementary figure 3b). This expression profile underscores the multifaceted capability of NK cells to recognize and eliminate cancerous cells, emphasizing their potent cytotoxic potential and readiness to contribute to the anticancer immune response. Collectively, these findings highlight the enhanced functionality of Day 13 NK cells, which are equipped with critical markers for effective tumor targeting and immune system interactions.

**Figure 3:**
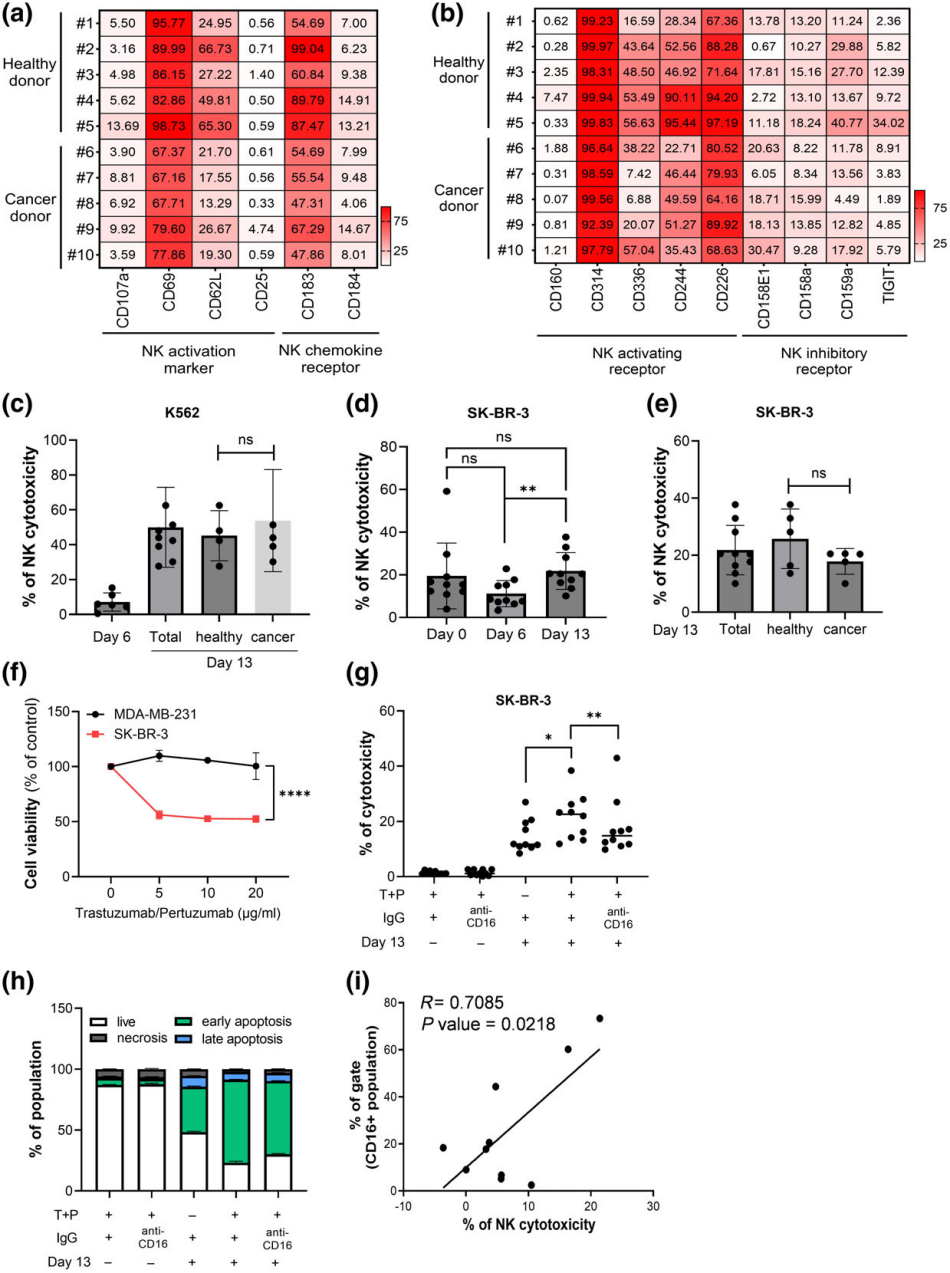
Analysis of NK cell activation and receptor expression, alongside their cytotoxic potential. **(a, b)** Gating percentages displayed within each cell to provide a detailed quantification of receptor expression levels within the CD56^+^ NK cell population. **(a)** Heatmap representation of flow cytometry‐based quantification of *NK* activation markers (CD107a, CD69, CD62L, CD25) and chemokine receptors (CD183, CD184) in the expanded NK cell population at Day 13. **(b)** Heatmap analysis of NK activating receptors (CD19b, CD314, CD335, CD244, CD226) and inhibitory receptors (CD160, CD159a, TIGIT) in the expanded *NK* cell population. **(c–e)** Explores the cytotoxic efficiency of expanded NK cells against the K562 cell line through an LDH assay conducted in triplicate at an effector:target (E:T) ratio of 20:1. The assay, carried out in duplicate with NK cells expanded from various donors (*n* = 9, including 4 healthy donors and 5 breast cancer patients), reveals no significant difference in the ability to kill K562 cells between NK cells from healthy donors and those from breast cancer patients, presenting an insight into the uniform cytotoxic capability of expanded NK cells irrespective of the donor's health status. ns, not significant. **(f)** Impact of monoclonal antibody treatments on HER2‐positive SK‐BR‐3 and triple‐negative MDA‐MB‐231 breast cancer cell lines over a 6‐day period. While trastuzumab and pertuzumab demonstrated no effectiveness against MDA‐MB‐231 cells, a significant enhancement in cytotoxic response was observed in SK‐BR‐3 cells when treated with a combination of trastuzumab and pertuzumab. **(g)** Depicts the enhanced cytotoxic effect observed when Day 13 NK cells are cotreated with trastuzumab and pertuzumab against SK‐BR‐3, illustrating the increased potency of this therapeutic combination. This panel also shows the significant reduction in cytotoxicity following the blockade of CD16, a key receptor in Antibody‐Dependent Cellular Cytotoxicity (ADCC), thereby emphasizing the importance of the CD16‐mediated ADCC pathway. **(h)** Through apoptosis analysis, this panel confirms the enhanced induction of cancer cell apoptosis by Day 13 NK cells compared to Day 0, and further illustrates the synergistic increase in apoptosis when NK cells are combined with SK‐BR‐3 on Day 13. This effect significantly diminishes with CD16 blockade, underscoring CD16's crucial role in mediating apoptosis. **(i)** Establishes a positive correlation between the CD16+ population of NK cells and the elimination of SK‐BR‐3 cancer cells on Day 13 for each donor, substantiating the critical role of the CD16‐mediated ADCC pathway, especially in combination with monoclonal antibodies targeting HER2‐positive breast cancer. This comprehensive analysis delineates the stages of NK cell maturation and their synergistic interaction with antibody‐based therapies, offering insights into the mechanisms enhancing their anti‐tumor efficacy. Statistical significance between compared groups is indicated as follows: ns = not significant, *P < 0.05, **P < 0.01.

To provide a quantitative comparison of NK receptor expression patterns, we incorporated heatmap analyses (Figure 3c, d). The activation and chemokine receptor panel (Figure 3a) demonstrated high expression of CD69 and CD62L, consistent with their opt‐SNE distribution. Notably, CD183 (CXCR3) appeared less prominently saturated in the opt‐SNE plot, but this was not due to a lack of expression. Instead, the heatmap data confirmed that highly saturated opt‐SNE regions (≥ 10^9^ expression level) appear red, whereas regions with lower expression are visually less prominent. This suggests that CD183 expression is present but distributed differently in intensity compared to other markers. In addition to activation receptors, we examined the expression of NK inhibitory receptors, including CD158e, CD158b, CD159a and TIGIT (Figure 3b). These receptors modulate NK cell activity by preventing excessive immune responses. Compared to activating receptors, inhibitory receptors were expressed at lower levels, indicating that expanded NK cells retain a phenotype biased toward activation rather than inhibition. Given that NK cell expansion peaks at Day 13, we focused our analysis on this time point, where the purity and activation status of CD56^+^ NK cells are most representative of the final expanded population. As a result, early‐stage time points (Days 1 and 6) were not separately analyzed for activation markers, since the Day 13 data sufficiently capture the overall activation state of the expanded NK cells. Additionally, total NK cell expansion data (Supplementary figure 1a) further support the increase in CD56^+^ NK cell populations, demonstrating a robust enrichment of NK cells throughout the culture period.

Following the identification of key activation receptors on Day 13 NK cells, indicating their enhanced readiness for anticancer action, we further investigated the functional implications of these cellular characteristics. We assessed the cytotoxic efficiency of expanded NK cells against K562 cells using an LDH assay, conducted in triplicate at an E/T ratio of 20:1 on NK cells expanded from a diverse group of donors (*n* = 9), including four healthy individuals and five patients with breast cancer. To establish a meaningful reference for cytotoxic activity, we included NK‐92 cells as a control group instead of Day 0 PBMCs, as the latter contains a heterogeneous immune cell population, including T cells, making direct comparisons with Day 13‐expanded NK cells challenging. Figure 3c demonstrates that Day 13‐expanded NK cells exhibit strong antitumor effects, with cytotoxic activity exceeding that of NK‐92 cells. Additionally, to ensure reproducibility, NK cells from two donors were tested in three independent replicates, confirming the consistency of the observed cytotoxicity trends. The results revealed a consistent cytotoxic capability of NK cells from both donor groups, underscoring the potential of Day 13 NK cells, regardless of the donor's health status, to uniformly target and eliminate cancer cells (Figure 3c–e). This finding provides insight into the uniform and potent cytotoxic potential of NK cells postexpansion, reinforcing their role in the effective targeting and eradication of tumor cells.

### HER2‐positive breast cancer treatment: The synergistic potential of NK cells and antibody therapy

HER2 positivity in breast cancer represents a critical subtype that substantially affects patient demographics, making HER2 overexpression a key target for therapies, notably immunotherapies tailored for HER2‐positive cancers. The SK‐BR‐3 cell line, characterized by its HER2‐positive phenotype, exhibits elevated levels of HER2, which correlate with more aggressive forms of breast cancer. We aimed to evaluate the antitumor efficacy of NK cells across various stages: resting NK cells on Day 0, semifinished NK cells on Day 6, and expanded NK cells on Day 13 against the HER2^+^ SK‐BR‐3 breast cancer cell line using the LDH assay to gauge the potency of TSBio autologous cell therapy. Analysis across 10 donors revealed a peak median killing capacity on Day 13 (Figure 3f), indicating no significant difference between healthy and cancer‐afflicted donors (Figure 3g).

Next, we evaluated the synergistic antitumor effects of HER2‐targeted antibody therapy in combination with NK cells against SK‐BR‐3 breast cancer cells. Before assessing the combined effects of trastuzumab and pertuzumab with NK cells, we first examined their individual effects using an independent viability assay. HER2‐positive SK‐BR‐3 and triple‐negative MDA‐MB‐231 breast cancer cell lines were treated for 6 days to determine their response to monotherapy treatment. The results confirmed that trastuzumab significantly reduced SK‐BR‐3 cell viability at 10 and 20 μg mL^−1^, whereas pertuzumab alone had no significant effect (Supplementary figure 3c). We then assessed the combined effect of trastuzumab and pertuzumab. As expected, neither antibody had a significant impact on MDA‐MB‐231 cells. However, in SK‐BR‐3 cells, the combination of trastuzumab and pertuzumab significantly enhanced cytotoxicity at 10 and 20 μg mL^−1^ (Figure 3h). Specifically, when treated with trastuzumab alone, SK‐BR‐3 cell viability was approximately 60% at both 10 and 20 μg mL^−1^. In contrast, cotreatment with trastuzumab and pertuzumab further reduced cell viability to 50%, indicating a synergistic cytotoxic effect. Next, we examined the role of Day 13‐expanded NK cells in antibody‐dependent cellular cytotoxicity (ADCC) by cotreating SK‐BR‐3 cells with NK cells, trastuzumab and pertuzumab. The results revealed that NK cell‐mediated cytotoxicity was significantly enhanced in the presence of both antibodies, confirming an ADCC effect (Figure 3i). To further validate this ADCC mechanism, we performed a CD16 blockade assay, as CD16 is a key receptor‐mediating ADCC. Blocking CD16 in the trastuzumab + pertuzumab (T + P) group led to a significant reduction in NK cell cytotoxicity, confirming that the observed cytotoxicity was ADCC‐dependent. Moreover, comparing NK cell cytotoxicity between CD16‐blocked samples and IgG‐treated controls further demonstrated that CD16 blockade significantly diminished NK cell‐mediated cytotoxicity, verifying ADCC as the primary mechanism underlying the observed cytotoxic response (Figure 3g).

### Functional characterization of NK cells: Cytokine dynamics and cytotoxicity against tumor cells

**Figure 4:**
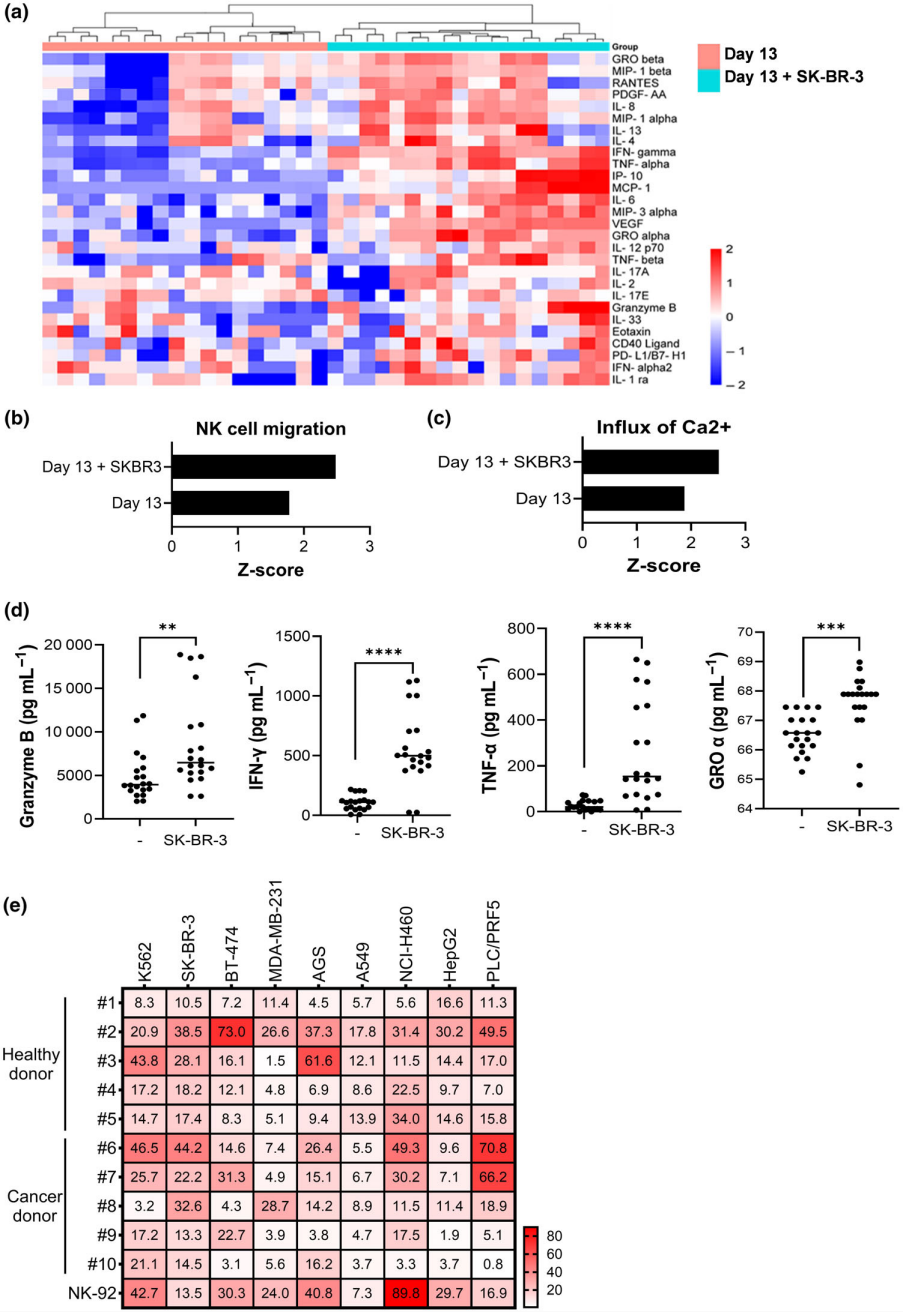
Cytokine dynamics and cytotoxic potential of Day 13 NK cells in response to tumor co‐culture. **(a)** Heatmap analysis depicting shifts in cytokine profiles observed after a 13‐day period of culturing *NK* cells both alone and in conjunction with cancer cells. **(b, c)** Ingenuity Pathway Analysis (IPA) results illustrating key biological pathways associated with NK cell activation. **(b)** NK cell migration significantly increased in Day 13‐expanded NK cells co‐cultured with SK‐BR‐3 breast cancer cells, compared to *NK* cells cultured alone. **(c)** Calcium influx (Ca^*2*+^) is also elevated in the presence of SK‐BR‐3, suggesting a potential link between NK cell migration and intracellular calcium signaling. These findings indicate that increased cell migration is associated with enhanced calcium influx, a known trigger of NFAT activation and NK cell maturation. **(d)** Increased secretion of key effector molecules such as granzyme B, IFN‐γ, TNFα and GROα by NK cells in response to cancer cell interaction. **(e)** Heatmap representation of the cytotoxic activity of donor‐derived NK cells against various solid tumor cell lines, highlighting donor‐specific variability in NK cell‐mediated cytotoxic responses. Statistical significance between compared groups is indicated as follows: **P < 0.01, ***P < 0.001, ****P < 0.0001.

In the context of our research on the enhanced anticancer effects of NK cells combined with HER2‐targeting antibodies, we delved deeper into the cytokine dynamics and molecular underpinnings of NK cell activation. Through a comparative analysis of cytokine expression in the culture supernatants of Day 13 NK cells, both alone and when cocultured with the SK‐BR‐3 cancer cell line, we aimed to identify cytokines pivotal for augmenting the anticancer activity of NK cells. Employing hierarchical cluster analysis to scrutinize the expression profiles of 28 distinct cytokines revealed notable upregulation of key cytokines in the cocultures of SK‐BR‐3 and Day 13 NK cells (Figure 4a).

Certain cytokines, such as TNF‐α, IFN‐γ and Granzyme B, exhibited a transient peak at Day 6, followed by a decline at Day 13. This dynamic pattern likely reflects a period of early immune activation driven by non‐NK immune cells (e.g. T cells and monocytes), which remain present at Day 6 and respond robustly to IL‐2 stimulation. By Day 13, the NK cell population is highly enriched, and cytokine secretion reflects a more regulated, NK cell‐dominant state. This pattern is consistent with previous NK cell differentiation studies, where cytokine profiles shift as NK cells mature and expand. To evaluate the robustness of the clustering structure, we performed silhouette analysis and cophenetic correlation assessment. The silhouette score was 0.254, indicating moderate intracluster similarity. The cophenetic correlation coefficient was 0.576, reflecting a moderate fit between the dendrogram structure and the pairwise distances of the data. Although these values suggest only modest clustering robustness, they support the observed donor‐dependent grouping trend shown in Figure 4a.

To further elucidate the molecular mechanisms underlying NK cell activation in response to tumor cells, we conducted Ingenuity Pathway Analysis (IPA) to compare cytokine profiles between Day 13 NK cells cultured alone and those cocultured with SK‐BR‐3 cancer cells. IPA results revealed a significant increase in NK cell migration upon coculture with SK‐BR‐3 cells (Figure 4b). While migration can be influenced by various mechanisms, additional analysis of cytokine and metabolic factors indicated that this response was not driven by inflammation or proliferation. Specifically, key inflammatory cytokines and glucose uptake levels remained unchanged, suggesting that enhanced NK cell migration was tumor recognition‐driven rather than a general immune response.

In Supplementary figure 3d, calcium influx is a well‐established trigger for NFAT activation, which plays a crucial role in NK cell functional maturation. Our analysis showed that NK cells cocultured with SK‐BR‐3 exhibited increased calcium uptake compared to NK cells cultured alone. Further supporting this activation profile, downstream signaling molecules associated with *NK* cell activation (including NFAT, IL‐15 and mTOR) were significantly upregulated (Supplementary figure 3d). These findings suggest that tumor‐induced calcium influx leads to NFAT activation, which in turn enhances NK cell function through multiple signaling pathways.

Taken together, these results provide mechanistic insights into how NK cells respond to tumor environments, demonstrating that their activation in the presence of cancer cells is driven by tumor recognition rather than inflammation or general proliferation.

To evaluate the therapeutic potential of Day 13‐expanded NK cells against solid tumors, we conducted a comprehensive cytotoxicity screening assay using NK cells derived from both healthy and cancer donors. The cytotoxic activity of these cells was compared to NK‐92, a widely used standard NK cell line in NK‐based immunotherapy. The heatmap representation (Figure 4e) illustrates the variability in cytotoxic responses across different donors and tumor types.

Among the tumor cell lines tested, SK‐BR‐3 (HER2^+^ breast cancer) exhibited the highest sensitivity to Day 13 NK cells. Notably, seven donors (#2, #3, #4, #5, #6, #7 and #8) demonstrated higher cytotoxic activity against SK‐BR‐3 compared to NK‐92. Similarly, PLC/PRF5 (liver cancer) showed strong responsiveness, with four donors (#2, #6, #7 and #8) surpassing NK‐92 in cytotoxic activity. A549 (lung cancer) also exhibited increased sensitivity to Day 13 NK cells, with three donors (#2, #3 and #5) displaying stronger cytotoxic responses than NK‐92. In addition, BT‐474 (HER2^+^ breast cancer), MDA‐MB‐231 (breast cancer) and AGS (gastric cancer) also demonstrated enhanced NK cell‐mediated cytotoxicity, with one donor (#2 for BT‐474, #8 for MDA‐MB‐231 and #3 for AGS) surpassing NK‐92 in cytotoxic efficiency. These results indicate a statistically significant difference compared to NK‐92, with *P* < 0.01. These findings highlight the donor‐specific variability in NK cell responses and suggest that certain individuals may generate more potent NK cells for tumor targeting.

In contrast, NK‐92 exhibited significantly higher cytotoxicity against NCI‐H460 (lung cancer) compared to all donor‐derived NK cells, suggesting that NK‐92 may be more effective for targeting this specific tumor type. Overall, these results indicate that Day 13‐expanded NK cells (TS‐NK) exhibit promising cytotoxic potential against solid tumors, particularly HER2‐positive breast cancer (SK‐BR‐3, BT‐474), breast cancer (MDA‐MB‐231), liver cancer (PLC/PRF5), lung cancer (A549) and gastric cancer (AGS) (Figure 4e). The observed variability in cytotoxic responses among donors further underscores the importance of personalized approaches in NK cell‐based immunotherapy.

Unlike NK‐92 cells, which require irradiation to prevent excessive proliferation, TS‐NK cells are derived from PBMCs and expanded *ex vivo*, eliminating the need for irradiation while maintaining full functionality *in vivo*. Furthermore, TS‐NK cells possess several advantages that enhance their clinical applicability. These expanded NK cells exhibit prolonged persistence *in vivo*, allowing for sustained anti‐tumor activity over time. Additionally, TS‐NK cells express CD16, enabling ADCC for effective tumor targeting, a feature absent in NK‐92 cells.

These findings emphasize the clinical potential of expanded NK cells in targeting solid tumors that are difficult to treat with conventional therapies. The strong cytotoxic activity observed against SK‐BR‐3 reinforces the feasibility of NK cell therapy for HER2‐positive cancers, particularly in combination with monoclonal antibodies. Moreover, TS‐NK cells demonstrate superior efficacy compared to NK‐92, positioning them as a highly promising candidate for clinical translation. Their ability to provide enhanced tumor targeting, sustained immune activity, and reduced risk of immune rejection further supports their potential application in NK cell‐based immunotherapy.

The observed cytokine profile suggests a robust activation of NK cells, characterized by enhanced cytolytic activity (granzyme B), pro‐inflammatory responses (IFN‐γ and TNFα),^21^, ^22^ and potential recruitment and modulation of other immune cells within the tumor milieu (GROα known as a CXCL1)^23^ (Figure 4d). This upsurge underscores the dynamic interplay between NK cells and cancer cells within the tumor microenvironment, highlighting how NK cells directly attack cancer cells as well as modulate the immunological context to reinforce their anticancer response. These findings further validate the therapeutic potential of NK cells in targeting HER2‐positive breast cancers and lay the groundwork for optimizing NK cell‐based immunotherapies by leveraging an intricate cytokine network.

## DISCUSSION

The efficacy of monoclonal antibody therapy can be enhanced by augmenting the function or number of NK cells through immunotherapy. Advances in precision medicine have allowed *in vitro* studies using patient‐derived cell lines to tailor treatments to the specific characteristics of each patient's cancer, potentially improving treatment outcomes. While *in vitro* models do not fully replicate the human tumor microenvironment, they are essential for initial assessments of NK cell activity, providing foundational data to guide subsequent *in vivo* studies. These models help in understanding the basic interactions and efficacy of NK cells against cancer cells, which is critical for further translational research.

This study aimed to differentiate and amplify NK cells from PBMCs of five healthy donors and five patients with breast cancer to verify the ADCC effect on HER2‐positive breast cancer. To ensure the purity of the NK cell therapy products manufactured in TSBio's GMP facility, minimization of CD3^+^ T cells, CD14^+^ monocytes, and CD19^+^ B cell contamination, and high purity of CD56^+^ or CD56^+^CD16^+^ *NK* cells were confirmed at various stages (Days 0, 6 and 13). In addition, changes in cytokine production in response to treatment have been examined in cell lines. The findings confirmed that using NK cells from healthy individuals as a control group was essential, as it provided a baseline for understanding the behavior of cancer‐derived NK cells without the need to separate CD3‐positive NK cells (NKT cells). This approach allowed the research to focus on the specific capabilities and enhancements of NK cells in targeting cancer cells effectively, demonstrating the critical role of a controlled comparative analysis in validating the anticancer activity of expanded NK cells.

Conducting multiplex cytokine analysis across varying culture periods of NK cells (Days 0 and 13) is pivotal for delineating dynamic shifts in their immunological profiles.^24^ This approach facilitated the identification of specific cytokines that are modulated during cell expansion. Such insights are instrumental in uncovering the underlying mechanisms that may bolster NK cell activation, proliferation and capacity to combat cancer. Analysis of cytokine patterns from 10 donors revealed diverse expression profiles of 28 cytokines across the culture period (Days 0, 6 and 13) that were not influenced by the donor type. By Day 13, NK cell efficacy indicators, granzyme B and IFN‐γ, along with IL‐17E, significantly increased, indicating enhanced activity and potential for combating cancer.^25^, ^26^ Understanding these changes may offer insights into NK immunotherapy pathways and guide biomarker development to predict treatment responses.

To elucidate the activation status of NK cells, focusing on the activation/chemokine receptor profiles, sheds light on the responsiveness of NK cells to inflammatory cues and their migratory aptitude toward the tumor locale. This is instrumental in understanding the readiness of cells to respond to and infiltrate the affected areas. The expression of NK cell‐activating receptors also shows offering a detailed view of the cytotoxic potential of the cells. These receptors are crucial for the identification and eradication of cancer cells, and their expression levels serve as critical measures of NK cell preparedness to confront and annihilate cancer targets. By integrating these analytical approaches, we aimed to thoroughly assess the functional capacity of NK cells and determine whether the cultivation process yielded NK cells enhanced in their anticancer prowess.^25^ Notably, CD69 is rapidly upregulated in NK cells upon activation and serves as an early indicator of activation. This CD69 upregulation suggests that NK cells actively participate in immune responses, highlighting their readiness for immune engagement.^27^ CD62L functions as a homing receptor, guiding cells toward lymphoid tissues, such as lymph nodes, and facilitating their migration and localization in areas critical for initiating immune responses.^28^ The choice of the SK‐BR‐3 cell line, known for its sensitivity to monoclonal antibodies and other anti‐HER2 targeted therapies, aimed to deepen our understanding of cancer biology and the nuances of treatment responses, including potential synergies with immunotherapeutics and immune checkpoint inhibitors. Furthermore, combining NK cells with SK‐BR‐3 cancer cells led to a significant upregulation of cytokines, explaining the molecular dynamics underlying NK cell activation in HER2‐positive breast cancer and highlighting their enhanced anticancer potential through complex cytokine‐mediated interactions. These findings emphasize the role of specific cytokines in strengthening NK cell cytotoxicity and the immune response against cancer and suggest pathways to target these mechanisms to improve NK cell‐based therapies.

Subsequent assessments of NK cell activation and their capability to effectively target cancer cells revealed significant upregulation of the activation markers CD62L and CD69 on NK cells, signifying their readiness for an immune response and migration into lymphoid tissues.^29^, ^30^ Moreover, the expression of various activating receptors on Day 13 underscores the enhanced ability of NK cells to recognize and destroy cancer cells, thus affirming their potential in cancer immunotherapy.

While our study demonstrates the effective expansion of NK cells in a 1 L culture bag system, it is essential to consider how culture volume and bioprocess conditions impact NK cell proliferation efficiency. In GMP‐scale expansion (50 L bioreactors), NK cells typically achieve > 1000‐fold expansion, whereas our 1 L system resulted in an average 32.6‐fold expansion. This discrepancy is not solely due to culture volume but reflects the influence of oxygenation, nutrient distribution and metabolic byproduct accumulation on NK cell proliferation. Future optimization efforts should focus on enhancing bioprocess conditions, including improving oxygen delivery, regulating cell density, and implementing automated metabolic waste management, rather than relying on direct volume scaling alone. Such refinements could significantly enhance NK cell yield and maintain consistent expansion efficiency in larger scale systems.

As we consider the implications and potential enhancements of our study on the efficacy of monoclonal antibody therapy augmented with NK cells, several limitations warrant further exploration. First, while our multiplex cytokine analysis has provided valuable insights into the cytokine profiles and activation markers such as CD62L and CD69, a more in‐depth mechanistic analysis is required to fully elucidate the cellular pathways and interactions that underpin these changes. Second, the current study has focused on the broader immunological profiles and activation states of NK cells, yet the complexity of the tumor microenvironment may necessitate a more detailed exploration of the interplay between NK cells and other immune cells within this context.

Our *in vitro* models, while not fully representative of the human tumor environment, provide essential preliminary data on NK cell activity. These studies are foundational for guiding *in vivo* research and understanding the basic interactions of NK cells with cancer cells. This study highlights the significant enhancement of NK cell anticancer activity when combined with HER2‐targeting antibodies, and particularly the role of cytokine dynamics in NK cell activation. Key cytokines were identified as being notably upregulated when NK cells were cocultured with SK‐BR‐3 cancer cells, suggesting their crucial role in augmenting NK cell function. This enhancement in activation was further validated by IPA, which showed that the presence of cancer cells significantly amplified NK cell activation independent of NK cell growth. These findings highlight the potential of leveraging specific cytokine pathways to optimize NK cell‐based immunotherapies for HER2‐positive breast cancer treatment. Building on this, research has delved into cytokine dynamics, revealing the key roles of cytokines in NK cell activation when combined with HER2‐targeting antibodies. This synergy between NK cells and antibodies, characterized by upregulated cytokine profiles and robust NK cell activation, validates the therapeutic potential of NK cells for targeting HER2‐positive breast cancers, and lays the foundation for optimizing NK cell‐based immunotherapy.

## METHODS

### Peripheral blood mononuclear cell (PBMC) isolation

This study was approved by the Institutional Review Board (IRB) of Ewha Womans University Mokdong Hospital (approval number: EUMC 2021‐12‐007‐010). Detailed information on the 10 donors (5 healthy individuals and 5 cancer patients) is provided in Table 2. Donor blood was collected under IRB‐approved protocols and processed under GMP‐compliant conditions in a facility licensed as a Management Business of Human Cells (No. 42) and an Advanced Regenerative Medicine Cell Processing Facility (No. 10) by the Korean Ministry of Food and Drug Safety. To ensure donor suitability, screening tests for hepatitis B, hepatitis C, AIDS, cytomegalovirus, syphilis and gonorrhea were conducted 1 week before blood collection, and blood was drawn only if the donor met the eligibility criteria. For PBMC isolation, 80 mL of fresh heparinized blood was collected, and buffy coat separation was performed using Ficoll‐Paque density gradient centrifugation. The isolated PBMCs were immediately used for NK cell expansion. This method ensured the enrichment of mononuclear cells while minimizing contamination with erythrocytes and granulocytes.

**Table 2:** Donor information.

Donor No.	Donor type	Year of birth	Male/Female	Breast cancer staging	
1	Healthy donor	1980	Female	—	
2	Healthy donor	1996	Female	—	
3	Healthy donor	1997	Female	—	
4	Healthy donor	1987	Female	—	
5	Healthy donor	1994	Female	—	
6	Cancer patient	1970	Female	1A	T1c N0
7	Cancer patient	1961	Female	0	Tis
8	Cancer patient	1958	Female	2A	T2 N0
9	Cancer patient	1970	Female	1A	T1b N0
10	Cancer patient	1977	Female	2B	T2 N1a

### NK cell expansion and quality control

PBMCs were isolated from 80 mL of fresh heparinized blood using density gradient centrifugation (Ficoll‐Paque PLUS, GE Healthcare, Chicago, IL, USA), yielding (2–8) × 10^7^ PBMCs per sample. On Day 0, 2 × 10^7^ PBMCs were seeded in 50 mL of NKCC‐1 medium (10% fetal bovine serum (FBS), Kohjin Bio, Japan) in T75 flasks precoated with NKCC‐c to enhance NK cell activation. Cells were cultured at 37°C with 5% CO_2_ for 6 days. On Day 6, 1.5–2 × 10^8^ activated PBMCs were transferred to a 1‐L culture bag containing 1000 mL of NKCC‐1 medium for large‐scale expansion. To mimic bioreactor conditions, cells were incubated under continuous gentle rocking (10–15 rpm), ensuring optimal oxygenation and nutrient distribution. On Day 13, > 2 × 10^9^ highly pure NK cells were harvested. The final NK cell product underwent cell count and viability assessment (trypan blue exclusion assay), phenotypic analysis (CD56^+^CD3^−^ purity via flow cytometry, Kaluza software) and sterility and mycoplasma contamination testing. Functional potency was evaluated using SK‐BR‐3 cells, a HER2‐positive breast cancer line, to assess natural cytotoxicity and ADCC in the presence of trastuzumab. A schematic diagram (Figure 1a) outlines the stepwise NK cell expansion process, illustrating the transition from small‐scale activation to large‐scale culture. Expanded NK cells were cryopreserved using 90% FBS and 10% dimethyl sulfoxide (DMSO) with a controlled‐rate freezing protocol to maintain viability. Cells were gradually cooled to −80°C before being transferred to liquid nitrogen (−196°C) for long‐term storage. Before use, frozen NK cells were rapidly thawed at 37°C, immediately diluted in prewarmed NKCC medium, and centrifuged to remove residual DMSO. The cells were then resuspended in fresh medium for subsequent culture or functional analysis.

### Characterization and maintenance of cancer cell lines for study

A diverse array of cancer cell lines was utilized in this study to ensure a broad representation of cancer types. All cell lines were sourced from the Korean Cell Line Bank (KCLB, Seoul, South Korea). The breast cancer cell lines used were SK‐BR‐3 (HER2^+^, KCLB no. 30030), BT474 (Luminal A, KCLB no. 60062) and MDA‐MB‐231 (TNBC, KCLB no. 30026). The liver cancer cell lines included HepG2 (KCLB no. 88065) and PLC/PRF/5 (KCLB no. 28024). Additionally, gastric cancer cell lines AGS (KCLB no. 21739), lung cancer cell lines A549 (KCLB no. 10185) and NCI‐H460 (KCLB no. 30177) and the leukemia cell line K562 (KCLB no. 10243) were also included in this study. For optimal growth, all cell lines were maintained in RPMI 1640 medium supplemented with 10% FBS, except for HepG2, which was cultured in Minimum Essential Medium (MEM) with 10% FBS. Cells were incubated in a humidified incubator at 37°C with 5% CO_2_ to simulate physiological conditions and ensure consistent cell growth. For long‐term preservation, all cell lines were frozen at a density of 1 × 10^6^ to 5 × 10^6^ cells mL^−1^ using Bambanker freezing medium (Nippon Genetics, Japan) as a cryoprotectant. Cells were gradually frozen at −80°C for 24 h before being transferred to liquid nitrogen (−196°C) for long‐term storage. Before experiments, frozen cells were rapidly thawed in a 37°C water bath, gently resuspended in fresh culture medium, and centrifuged at 300× *g* for 5 min to remove residual freezing medium.

### Reagents and antibodies

Trastuzumab (cat# A2007) and pertuzumab (cat# A2008) were acquired from Selleck Chemicals (Houston, TX, USA). The CytoTox 96® nonradioactive cytotoxicity assay kit, essential for evaluating cell cytotoxicity, was sourced from Promega (cat# G1780, Madison, WI, USA). For the cell viability assessment, a CCK‐8 assay kit was purchased from Dojindo (cat# CK04‐05, Kumamoto, Japan). The Dead Cell Apoptosis Kit with Annexin V‐FITC and propidium iodide for flow cytometry, which is pivotal for identifying apoptotic versus necrotic cells, was obtained from Invitrogen (cat# V13242, Carlsbad, CA, USA). The antibodies used for flow cytometry analysis and blocking experiments are summarized in Table 3. This table shows their specific targets, clones, conjugations, manufacturers and catalog numbers, ensuring that the experimental framework can be precisely replicated and validated by others in the field. This level of detail underscores the commitment to transparency and rigor in presenting the experimental methodologies.

**Table 3:** Antibody lists.

NK marker antibodies and multi‐color panel								Filter set
#	Cellular marker/antigen	Clone	Conjugation/fluorophore	Dilution	Supplier	Cat#	Excitation laser (nm)	BP
1	Mouse IgG1	MOPC‐21	FITC	1:25	BioLegend	400108	488	525/40
2	CD16	3G8				302006		
3	CD107a	H4A3				328606		
4	CD94	DX22				305504		
5	Mouse IgG1	MOPC‐21	PE			400112		585/42
6	CD69	FN50				310906		
7	CD160	BY55				341206		
8	Mouse IgG1	MOPC‐21	PE‐Cy5			400118		690/50
9	CD62L	DREG‐56				304808		
10	CD314/NKG2D	1D11				320844		
11	CD183/CXCR3	G025H7				353720		
12	CD336/NKp44	P44‐8				325116		
13	Mouse IgG2a	MOPC‐173	APC			400220	638	660/20
14	CD56/NCAM	MEM‐188				304610		
15	Mouse IgG2a	MOPC‐173	APC‐Cy7			400230		780/60
16	CD3	HIT3a				300318		
17	Mouse IgG1	MOPC‐21	Pacific Blue			400151	405	450/45
18	CD14	63D3				367122		
19	CD25	BC96				302627		
20	CD244/2B4	C1.7				329524		
21	Mouse IgG1	MOPC‐21	BV510			400172		525/40
22	CD19	HIB19				302242		
23	CD184/CXCR4	12G5				306536		
24	CD226/DNAM‐1	11A8				338330		

### Cytotoxicity assay to evaluate the anticancer efficacy of HER2‐targeted therapies

To evaluate the anticancer efficacy of HER2‐targeted therapies, trastuzumab and pertuzumab were tested individually and in combination in HER2‐positive breast cancer cell lines (SK‐BR‐3) and a triple‐negative cell line (MDA‐MB‐231) to assess specificity. Cells were seeded at 5 × 10^3^ per 100 μL in 96‐well plates and treated with varying concentrations of the antibody drugs (5, 10 and 20 μg mL^−1^), with treatments applied every 2 days. Cell viability was measured after 6 days using the CCK8 assay according to the manufacturer's protocol (Dojindo), with the absorbance read at 480 nm. The optimal treatment condition identified was a combined therapy at a concentration of 10 μg mL^−1^ for both drugs, which was used in subsequent experiments to further investigate their combined anticancer effects, emphasizing the potential for synergistic activity against HER2‐positive breast cancer.

### Assessing NK cell‐mediated cytotoxicity against cancer cells using the lactate dehydrogenase (LDH) release assay

The cytotoxic activity of Day 13‐differentiated NK cells was evaluated using the CytoTox 96® nonradioactive cytotoxicity assay kit (Promega), which quantifies lactate dehydrogenase (LDH) release as a marker of cell lysis. Following the manufacturer's protocol, we measured NK cell‐mediated cytotoxicity against HER2‐positive breast cancer cell lines (SK‐BR‐3, BT474), triple‐negative breast cancer (MDA‐MB‐231), hepatocellular carcinoma (HepG2, PLC/PRF/5), gastric cancer (AGS) and lung cancer (A549, NCI‐H460), in addition to the hematologic cancer cell line (K562). All cancer cell lines were seeded in V‐bottom 96‐well plates at a density of 1 × 10^4^ cells/well. NK cells derived from 10 donors were added at an effector‐to‐target (E/T) ratio of 20:1. To evaluate antibody‐dependent cellular cytotoxicity (ADCC), NK cells were pretreated with a blocking antibody (anti‐CD16, cat# 302002, BioLegend, San Diego, CA, USA) at 1 μg mL^−1^ for 30 min prior to coculture. For HER2‐expressing targets (SK‐BR‐3, BT474), the monoclonal antibodies trastuzumab and pertuzumab (T + P) were added to assess their role in enhancing ADCC. Following centrifugation at 400× *g* for 4 min, the coculture was incubated at 37°C in 5% CO_2_ for 4 h. The LDH released into the supernatant, indicating cytotoxicity, was quantified by measuring absorbance at 490 nm using a microplate reader. Cytotoxicity was calculated based on the manufacturer's protocol to ensure reproducibility and accuracy in assessing NK cell‐mediated cancer cell killing across multiple tumor types.

### Apoptosis assay using annexin V FITC and propidium iodide

Apoptosis of SK‐BR‐3 cells, induced by NK cells derived from PBMCs on Days 6 and 13 from 10 donors, was meticulously analyzed using the Dead Cell Apoptosis Kit with Annexin V FITC & Propidium Iodide for Flow Cytometry (Invitrogen). Initially, to ensure the specific detection of cancer cell apoptosis, SK‐BR‐3 cells were labeled with 5 μM CellTrace™ Violet Cell Proliferation Kit (Invitrogen, Cat# C34557) for 20 min at 37°C in 5% CO_2_. Postlabeling, cells were centrifuged at 300 × *g* for 2 min, washed twice with PBS, and subsequently seeded at a density of 1 × 10^4^ cells per 50 μL in a V‐bottom 96‐well plate. To evaluate the ADCC of NK cells, a blocking antibody (anti‐CD16) was initially pretreated at a concentration of 1 μg mL^−1^ for 30 min, followed by the secondary treatment with trastuzumab and pertuzumab (each at 10 μg mL^−1^) for another 30 min. NK cells were then added to achieve an E/T ratio of 20:1, followed by centrifugation at 400× *g* for 4 min, and incubated for 4 h at 37°C in a 5% CO_2_ environment. Post co‐culture, cells were stained with 10 μL of Annexin V FITC and 2 μL of PI for 15 min in preparation for flow cytometry, facilitating the detailed assessment of the apoptotic stages, including necrosis, early apoptosis and late apoptosis, in accordance with the manufacturer's protocol.

### Immunophenotyping of NK cells via flow cytometry analysis

Flow cytometry was performed to characterize NK cell immunophenotypes. Cryopreserved NK cells were thawed at 37°C, resuspended in NKCC medium at 1 × 10^7^ cells mL^−1^, and incubated with Fc Receptor Binding Inhibitor (Invitrogen, cat# 14‐9161‐73, 1 μL per 50 μL) for 20 min at 4°C to reduce nonspecific antibody binding. After centrifugation at 400× *g* for 5 min, cells were resuspended in Flow Cytometry Staining Buffer (Invitrogen, cat# 00‐4222‐26) and plated at 1 × 10^5^ cells/well in a 96‐well plate (50 μL/well). Cells were then incubated with a panel of fluorescently labeled antibodies for 30 min at 4°C. Following incubation, cells were washed twice with PBS before proceeding to flow cytometric analysis. Flow cytometry was conducted using a CytoFLEX and MoFlo Astrios flow cytometer (Beckman Coulter), with daily quality control (QC) verification via CytExpert's QC module to ensure consistent instrument performance. Data acquisition was performed using CytExpert software (Beckman Coulter), and fluorescence compensation was applied using single‐stained controls to minimize spectral overlap. Unstained and isotype controls were used to define positive and negative gating thresholds. For data analysis, Kaluza software (v2.2, Beckman Coulter) was used to exclude doublets, dead cells and nonlymphoid populations before identifying NK cell subsets. Live, single NK cells were gated based on CD56^+^CD16^+^ expression, allowing for the exclusion of CD3^+^ T cells, CD14^+^ monocytes and CD19^+^ B cells (Supplementary figure 1c). To comprehensively assess NK cell phenotypes, three distinct antibody panels were utilized. The identity panel included markers for major immune cell lineages, including CD3, CD14, CD16, CD19 and CD56, to distinguish NK cells from other hematopoietic populations. The activation and chemokine receptor panel consisted of CD25, CD62L, CD69, CD107a, CD183 and CD184, allowing for the evaluation of NK cell activation states and migratory potential. The activation receptor panel included CD94, CD160, CD244, CD226 (DNAM‐1), CD314 (NKG2D) and CD336 (NKp44), providing insight into NK cell‐mediated cytotoxicity and immune modulation. For high‐dimensional analysis, CytoBank software (Beckman Coulter, CA, USA) was used to perform dimension reduction, enabling visualization of cell clustering patterns based on phenotypic similarities.

### Cytokine profiling via multiplex Array analysis

Cytokine secretion by NK cells from 10 donors was assessed at Days 0, 6, 13 and following coculture of Day 13 NK cells with SK‐BR‐3 cancer cells. For coculture, 2 × 10^5^ NK cells and 1 × 10^4^ SK‐BR‐3 cells were combined at an E/T ratio of 20:1 in 50‐μL volumes. Fresh media was replaced, and the cells were incubated for 4 h. After centrifugation (300 × *g*, 1 min), the supernatant was collected from NK cell‐only cultures, cocultures, and SK‐BR‐3 controls for comparison. Cytokine analysis was outsourced to LABISKOMA (Seoul, South Korea), where quantification was performed using a Luminex 200 multiplex bead array system with streptavidin‐PE fluorescence detection. Cytokine levels were measured using a Luminex‐based multiplex bead array assay kit (manufacturer, catalog number, if available), following the manufacturer's protocol. Fluorescence intensity was converted to cytokine concentration using a standard curve generated from serial dilutions of known cytokine standards included in each run. A full list of the 46 cytokines analyzed, including the assay detection range and sensitivity for each analyte, is provided in Table 4. The detection limits for five key cytokines were: Granzyme B (1.04 pg mL^−1^), IFN‐*γ* (0.062 pg mL^−1^), TGF*α* (0.93 pg mL^−1^), IL‐17E (1.95 pg mL^−1^) and GRO*α* (25.2 pg mL^−1^). A total of 46 cytokines were analyzed and, among them, 28 cytokines with *P*‐values < 0.05 were selected for hierarchical clustering analysis. Cytokine expression patterns were analyzed using hierarchical clustering (R, ComplexHeatmap package) and principal component analysis (PCA) (Perseus v2.1.3.0). Graphical visualization was generated in GraphPad Prism (v10.3.1). Statistical analysis was conducted using one‐way ANOVA with Tukey's multiple comparison test to assess differences in cytokine secretion across experimental conditions.

**Table 4:** List of cytokine multiplex analytes.

#	Analyte	Standard curve (pg mL^−1^)	Sensitivity (pg mL^−1^)	#	Analyte	Standard curve (pg mL^−1^)	Sensitivity (pg mL^−1^)
1	CCL2/JE/MCP‐1	4.5–3300	0.6	24	IL‐2	3.4–2500	0.25
2	CCL3/MIP‐1 alpha	13.7–3340	1.58	25	IL‐3	3.4–2500	2.63
3	CCL4/MIP‐1 beta	82.3–20 000	18.4	26	IL‐4	3.4–2500	0.07
4	CCL5/RANTES	247–180 000	40.7	27	IL‐5	6.2–4500	0.31
5	CCL11/Eotaxin	31.6–23 000	1.81	28	IL‐6	8.6–6300	0.38
6	CCL19/MIP‐3 beta	3.9–2850	0.6	29	IL‐7	3.3–2400	0.18
7	CCL20/MIP‐3 alpha	2.2–1600	1.05	30	IL‐8/CXCL8	1.8–1300	0.36
8	CD40 Ligand/TNFSF5	480–350 000	110.2	31	IL‐9	412–300 000	37.1
9	CXCL1/GRO alpha/KC/CINC‐1	57.6–14 000	25.2	32	IL‐10	61.1–44 560	5.47
10	CXCL2/GRO beta/MIP‐2/CINC‐3	9.9–7200	0.83	33	IL‐12 p70	18.5–13 500	2.39
11	CXCL10/IP‐10/CRG‐2	2.5–1800	0.16	34	IL‐13	28.4–20 700	3.39
12	EGF	6.7–4880	1.21	35	IL‐15	2.3–1700	0.26
13	FGF basic/FGF2/bFGF	9.6–7000	4.95	36	IL‐17/IL‐17A	8.1–5900	1.09
14	Flt‐3 Ligand/FLT3L	21.9–16 000	3.09	37	IL‐17E/IL‐25	17.8–13 000	1.95
15	G‐CSF	9.4–6880	0.62	38	IL‐33	8.9–6500	1.92
16	GM‐CSF	16.5–12 000	1.56	39	Lymphotoxin‐alpha/TNF‐beta	0.8–600	0.196
17	Granzyme B	7.5–5500	1.04	40	PD‐L1/B7‐H1	5.9–4320	0.532
18	IFN‐alpha 2/IFNA2	4.1–3000	0.29	41	PDGF‐AA	6.6–4800	0.86
19	IFN‐beta	2.7–2000	0.31	42	PDGF‐AB/BB	6.9–5000	0.23
20	IFN‐gamma	0.5–380	0.062	43	TGF‐alpha	8.8–6400	0.93
21	IL‐1 alpha/IL‐1F1	9.9–7200	0.41	44	TNF‐alpha	11–8000	0.62
22	IL‐1 beta/IL‐1F2	3.4–2500	0.25	45	TRAIL/TNFSF10	24–17 500	2.32
23	IL‐1ra/IL‐1F3	11–8000	5.98	46	VEGF	6.4–4700	1.17

### Quantification and statistical analysis

Data collection and analysis were performed using GraphPad Prism version 9.4.1. All data are expressed as the mean ± standard error of the mean. Statistical analyses were conducted using Student's *t‐*test and the Mann–Whitney *U‐*test for data that did not follow a normal distribution. Statistical significance levels are represented as ns (not significant), *P < 0.05; **P < 0.05; ***P < 0.01 and ****P < 0.001. Sample sizes were not predetermined using statistical methods but were similar to those commonly used in the field.

## CONCLUSION

This study demonstrates significant advancements in NK cell‐based immunotherapy, particularly by highlighting the potential of autologous NK cell therapies that do not require the depletion of CD3^+^ NKT cells. By preserving NKT cells during the *ex vivo* expansion of NK cells derived from cancer patients, we developed a simplified, cost‐effective process without compromising the therapeutic efficacy of NK cells. This approach not only reduces manufacturing complexity and production time but also potentially enhances immune responses by maintaining the synergistic interactions between NK and NKT cells.

Our findings indicate that NK cells derived from both healthy donors and breast cancer patients exhibit strong anticancer activity and mediate potent ADCC when combined with HER2‐targeting antibodies. The observed efficacy in targeting both hematologic and solid tumors reinforces the versatility of NK cell‐based immunotherapy. Furthermore, the integration of NK cells with monoclonal antibody treatments offers a promising strategy to enhance tumor‐specific cytotoxicity, particularly for HER2‐positive malignancies.

Future studies should focus on personalizing NK cell‐based therapies by considering the individual patient's immune profile and the complex interplay between NK and NKT cells. Tailoring these therapies to the tumor microenvironment and cytokine signaling pathways may further enhance clinical outcomes. Additionally, our analysis of cytokine dynamics during NK cell expansion suggests that strategic cytokine modulation could serve as a key approach to optimizing NK cell functionality.

The integration of NK cell‐based therapies with existing immunotherapeutic strategies holds significant potential for improving cancer treatment outcomes. By building upon these foundational insights, we aim to refine and expand the applications of NK cell therapies, ultimately striving to enhance survival rates and improve the quality of life for patients with cancer.

## AUTHOR CONTRIBUTIONS

**Eun Hee Han:** Conceptualization; data curation; formal analysis; funding acquisition; investigation; methodology; project administration; resources; software; supervision; validation; visualization; writing – original draft; writing – review and editing. **Jin Young Min:** Formal analysis; methodology; software; validation; visualization. **Tae Kyung Ko:** Data curation; formal analysis; investigation; methodology; validation; visualization. **Hye Min Kim:** Data curation; formal analysis; investigation; methodology; validation. **Hae Won Jung:** Data curation; methodology; validation. **Cha Ok Yim:** Conceptualization; data curation; project administration; supervision; validation; writing – review and editing.

## CONFLICT OF INTEREST

The authors declare that this study was funded by TSBio. The funder was involved in the study design; collection, analysis and interpretation of data; writing of this report; and the decision to submit the article for publication. This financial relationship can be construed as a competing interest.

## HUMAN ETHICS AND CONSENT TO PARTICIPATE

This study involved the use of human peripheral blood mononuclear cells (PBMCs) derived from healthy donors and patients with breast cancer. All procedures performed in studies involving human participants were in accordance with the ethical standards of the institutional and/or national research committee and with the 1964 Helsinki declaration and its later amendments or comparable ethical standards. This study was approved by the Institutional Review Board (IRB), with IRB approval number [EUMC 2021‐12‐007‐010].

## Supporting information


Supplementary figures 1–3

## Data Availability

The data that support the findings of this study are openly available on Figshare at https://doi.org/10.6084/m9.figshare.27178935.
