# Supplementary material for: Characterization of ex vivo expanded natural killer cells for cancer immunotherapy
Source: Immunol Cell Biol. 2025 Jun 18;103(7):664–82. doi: 10.1111/imcb.70038 (PMC12392702; doi:10.1111/imcb.70038)
Supplement: Supplementary file 1 — Supplementary figures 1–3 [file IMCB-103-664-s001.docx]

Supporting Information

**Characterization of ex vivo expanded natural killer cells for cancer immunotherapy**

Jin Young Min ^1,†^, Tae Kyung Ko ^2,†^, Hye Min Kim ^1,3^, Hae Won Jung ^1,3^, Cha Ok Yim ^2,^* and Eun Hee Han ^1,3,^*

^1^ Biopharmaceutical Research Center, Ochang Institute of Biological and Environmental Science, Korea Basic Science Institute (KBSI), Cheongju 28119, South Korea

^2^ TS BIO ALL Co., Ltd, Seoul 08389, South Korea

^3^ Korea University of Science and Technology (UST), Daejeon, 34113, South Korea

^†^ These authors contributed equally to this work.

**Correspondence:** E-mail: [coyim@tsvbio.com](mailto:coyim@tsvbio.com); [heh4285@kbsi.re.kr](mailto:heh4285@kbsi.re.kr)

**Supplementary figure 1.** Expansion efficiency and phenotype comparison of NK cells during culture. **(a)** Total NK cell counts at days 0, 6 and 13 across multiple donors, illustrating the progressive expansion of NK cells over time. **(b)** Mean fold expansion of NK cells at each time point, calculated relative to the initial seeding density, demonstrating the effectiveness of the expansion protocol. **(c)** Flow cytometry gating strategy for NK cell population analysis. Representative flow cytometry plots illustrate the sequential gating strategy used to identify NK cell subsets. Initially, dead cells and doublets were excluded to ensure data accuracy. To define positive and negative gating thresholds, unstained controls and isotype controls were incorporated. NK cells were identified based on CD56⁺CD16⁺ expression, while CD3⁺ T cells, CD14⁺ monocytes and CD19⁺ B cells were excluded from the analysis. The results shown are from donor #5, which was selected as a representative sample due to consistency across all donor samples. **(d)** opt-SNE visualization of NK cell phenotypes from multiple donors. This figure addresses the limitation of **Figure 1d** by displaying the phenotypic landscape of CD16⁺ and CD56⁺ populations in expanded NK cells from multiple donors, reinforcing the consistency of our findings. Using dimensionality reduction (opt-SNE) analysis generated with CytoBank software, this visualization compares NK cell phenotypic patterns between healthy donors and cancer patient donors at day 13. **(e)** Comparison of CD56⁺, CD16⁺ and CD56⁺/CD16⁺ NK cell populations between healthy donors and cancer patient donors on day 13. Flow cytometry analysis revealed no statistically significant differences (**ns**) in the frequencies of these NK cell subsets between the two groups. These results indicate that NK cell expansion yields comparable phenotypic distributions regardless of donor health status, reinforcing that the expansion process does not introduce major differences in NK cell surface marker expression.

**Supplementary figure 2.** Principal component analysis and hierarchical clustering of cytokine profiles during NK cell expansion. **(a)** Heatmap analysis of cytokine profiles at days 0, 6 and 13, excluding samples with undetectable values. Data were analyzed from two replicates using hierarchical clustering based on the concentration levels of 28 distinct cytokines in the culture medium. The clustering reveals distinct segregation of samples, reflecting changes in cytokine expression patterns over time. **(b)** Principal component analysis (PCA) of cytokine profiles at days 0, 6 and 13, illustrating significant differences in cytokine secretion patterns across time points (ANOVA, P < 0.0001). The separation of samples by time point highlights the progressive shifts in cytokine dynamics throughout NK cell expansion.

**Supplementary figure 3.** Analysis of NK cell activation and receptor expression and synergistic effects of trastuzumab and pertuzumab on breast cancer cell viability over a 6-day period. **(a)** opt-SNE visualization illustrating the co-expression of the activation marker CD69 and the homing marker CD62L within the CD56⁺ NK cell population on day 13, underscoring the cells' readiness for activation and their potential to infiltrate tumors. **(b)** Expression profiles of key activating receptors—CD94, CD160, CD244, CD226 (DNAM-1), CD314 (NKG2D) and CD336 (NKp44)*—*within the CD56⁺ subset, demonstrating the cells' enhanced capability for recognizing and destroying cancer cells, thereby emphasizing their pivotal role in orchestrating an effective anticancer immune response. **(c)** This figure complements **Figure 4c** by illustrating the combined effects of monoclonal antibody treatments on HER2-positive SK-BR-3 *a*nd triple-negative MDA-MB-231 breast cancer cell lines. Cell viability was assessed after treatment with trastuzumab or pertuzumab alone at 10 and 20 μg mL^–1^, or in combination. **(d)** Predictive expression analysis of *NK* cell activation markers. (Left) Under identical level of D-glucose and cytokine conditions, increased NK cell migration correlates with enhanced activation. (Right) Calcium influx is a well-known trigger of NFAT signaling, which plays a crucial role in *NK* cell maturation. Increased expression of IL-15 and mTOR further supports the metabolic and functional progression of NK cells. Data are presented as Z-scores, comparing day 13 NK cells with and without SK-BR-3 co-culture.
